# A 3D Alginate–Gelatin Co-Culture Model to Study Epithelial–Stromal Interactions in the Gut

**DOI:** 10.3390/gels12010070

**Published:** 2026-01-13

**Authors:** Paraskevi Tselekouni, Mansoureh Mohseni-Garakani, Steve Papa, Seong Yeon Kim, Rita Kohen Avramoglu, Michael R. Wertheimer, Abdellah Ajji, Peter L. Lakatos, Derek H. Rosenzweig

**Affiliations:** 1 Department of Surgery, McGill University, Montreal, QC H3A 0G4, Canadamansoureh.mohseni@mail.mcgill.ca (M.M.-G.);; 2 Department of Medicine, Division of Gastroenterology, McGill University, Montreal, QC H3A 0G4, Canadarita.kohen@muhc.mcgill.ca (R.K.A.); 3 Department of Engineering Physics, Polytechnique Montreal, Montreal, QC H3T 1J4, Canada; 4 Department of Chemical Engineering, Polytechnique Montreal, Montreal, QC H3T 1J4, Canada; 5 Department of Internal Medicine and Oncology, Semmelweis University, 1428 Budapest, Hungary; 6 Surgical & Interventional Sciences Program, The Research Institute of McGill University Health Centre, Montreal, QC H4A 3J1, Canada; 7 Department of Orthopedic Surgery, University of Connecticut School of Medicine, Farmington, CT 06032, USA

**Keywords:** hydrogel scaffold, 3D co-culture model, intestinal epithelium, epithelial–stromal interactions, electrospun scaffold, inflammatory bowel disease, LPS stimulation, anti-inflammatory response

## Abstract

Inflammatory bowel disease (IBD) arises from chronic dysregulation at the epithelial–stromal interface, creating a need for in vitro systems that better capture these interactions. In this study, we developed a 3D co-culture platform in which HT-29 intestinal epithelial cells and IMR-90 fibroblasts are embedded within an alginate–gelatin hydrogel, alongside a complementary interface model using a plasma-treated electrospun mesh to spatially compartmentalize stromal and epithelial layers. We first assessed metabolic activity, viability, and proliferation across several epithelial-to-fibroblast ratios and identified 1:0.5 as the most supportive of epithelial expansion. The A1G7 hydrogel maintained high viability (>92%) and sustained growth in all mono- and co-cultures. To evaluate inflammatory competence, models were stimulated with lipopolysaccharide (LPS), administered either within the hydrogel or through the culture medium. LPS exposure increased TNF-α and IL-1β secretion in both configurations, with the magnitude of the response depending on the delivery route. Treatment with dexamethasone consistently reduced cytokine levels, confirming the model’s suitability for pharmacological testing. Together, these results demonstrate that the alginate–gelatin system provides a reproducible epithelial–stromal platform with quantifiable inflammatory readouts, offering a practical foundation for mechanistic studies and early-stage screening of anti-inflammatory therapeutics in IBD.

## 1. Introduction

Inflammatory Bowel Disease (IBD) is a chronic gastrointestinal disorder characterized by persistent inflammation of the digestive tract. It encompasses two primary conditions, Crohn’s disease (CD) and ulcerative colitis (UC), which exhibit distinct pathophysiological manifestations yet share common inflammatory pathways and disease progression patterns [1]. In North America, IBD now affects more than 2.5 million people (approximately 0.8% of the population as of 2024), and projections indicate that this burden will continue to grow in the coming decades [2]. Canada similarly ranks among the countries with the highest prevalence worldwide. An estimated 322,600 Canadians (0.82% of the population) were living with IBD in 2023, and this number is expected to rise to about 470,000 by 2035, representing nearly 1.1% of the population [3]. Although IBD was historically more common among individuals of European ancestry, recent epidemiological studies document an increasing incidence across diverse ethnic groups globally, reaching corresponding incidence values in 2nd generation immigrants to those of the native ethnic groups [4]. The disease is most frequently diagnosed in adolescents and young adults, and a steep rise is reported in children [5,6].

Despite advancements in therapeutic interventions, including corticosteroids, immunomodulators, and biologics, many patients experience heterogeneous responses, primary non-response, or secondary loss of efficacy over time, necessitating the development of personalized medicine approaches [7,8,9]. The multifactorial nature of IBD pathogenesis, which is driven by complex interactions between genetic predisposition, mucosal immune dysregulation, epithelial barrier dysfunction, and gut microbiota alterations, highlights the urgent need for advanced, physiologically relevant in vitro models that accurately replicate the human intestinal microenvironment to enable disease modeling and drug screening [10,11,12].

The intestinal stroma plays a critical role in maintaining tissue homeostasis and modulating inflammatory responses. Among stromal components, fibroblasts are key mediators of the epithelial–stromal interface, contributing to tissue remodeling, ECM deposition, and immune regulation in IBD [13,14,15]. Under homeostatic conditions, fibroblasts secrete structural proteins and growth factors, supporting epithelial renewal and barrier function [13,16]. However, during chronic inflammation in IBD, fibroblasts become highly reactive, leading to excessive ECM remodeling, fibrosis, and sustained production of inflammatory cytokines such as tumor necrosis factor-alpha (TNF-α), interleukin-6 (IL-6), and transforming growth factor-beta (TGF-β) [14,15]. These activated fibroblasts interact dynamically with epithelial cells, immune cells, and microbial signals, thereby perpetuating chronic inflammation and fibrotic remodeling [14,17]. Given their dual role in both tissue repair and inflammation, fibroblasts are crucial for accurately recapitulating the IBD microenvironment and evaluating novel therapeutic strategies targeting epithelial–stromal interactions [15,18]. These complex epithelial–stromal dynamics are difficult to reproduce using conventional in vitro approaches, which limits their utility for studying IBD pathophysiology.

Conventional in vitro models, including 2D monolayer cultures of intestinal epithelial cells, have provided fundamental insights into IBD pathophysiology, yet they are limited in their ability to recapitulate the biochemical and spatial complexity of the intestinal tissue architecture. These models fail to incorporate the intricate epithelial–stroma interactions, cytokine-mediated inflammation, and extracellular matrix (ECM) composition observed in vivo that are essential for disease progression and therapeutic response evaluation [19,20,21]. To overcome these challenges, recent advancements in bioengineering have facilitated the development of biomaterial-based 3D culture systems, such as hydrogels, organoids, and scaffold-based systems [15,22,23]. Among these, alginate-gelatin hydrogels have emerged as an optimal biomaterial scaffold for recreating the IBD microenvironment due to their ability to support cell viability, proliferation, and differentiation [24,25]. Alginate provides a biocompatible, mechanically tunable scaffold that mimics ECM properties, while gelatin enhances cell adhesion, promoting epithelial–stromal and epithelial–matrix interactions [26,27,28]. A complementary engineering strategy involves combining the alginate–gelatin hydrogel with a polymeric nanofibrous scaffold to create a two-compartment interface between the stroma and epithelium. This configuration more closely mimics the physiological microenvironment and provides a more relevant model for studying disease complexity [29,30,31]. Herein, the alginate–gelatin hydrogel was selected as a well-established and reproducible matrix, not to introduce a novel material formulation, but to serve as a robust and controllable biological platform for investigating epithelial–stromal interactions and inflammatory responses relevant to IBD, where control is achieved through defined composition, crosslinking strategy, and spatial organization rather than through material innovation.

In this study, we present a novel 3D co-culture system comprising intestinal epithelial cells and fibroblasts encapsulated within an alginate–gelatin hydrogel to model epithelial–stromal interactions, cytokine-mediated inflammation, and therapeutic responses in IBD. The optimal ratio of fibroblasts to epithelial cells for best modeling of gut inflammatory responses was determined within the 3D hydrogel culture. Additionally, we employed an advanced 3D in vitro interface model designed to more accurately recapitulate the complex stroma–epithelial interactions characteristic of IBD. This model is based on a plasma-treated nanofibrous electrospun mesh seeded with fibroblasts to represent the stromal compartment, overlaid with an alginate–gelatin hydrogel embedding intestinal epithelial cells. The resulting configuration forms a two-compartment tissue interface that closely resembles the physiological architecture of the intestinal mucosa.

To evaluate the system’s physiological relevance and drug screening potential, we assessed the model’s response to pro-inflammatory stimuli and subsequent therapeutic intervention with anti-inflammatory agents. We hypothesize that this 3D co-culture system will provide a clinically relevant platform for studying IBD pathophysiology and serve as a screening tool for anti-inflammatory therapeutics. By bridging the gap between traditional 2D cultures and the in vivo microenvironment, this approach offers a scalable, reproducible system for investigating personalized treatment strategies for IBD patients, ultimately contributing to the development of precision medicine in gastroenterology.

## 2. Results and Discussion

### 2.1. Characterization of 3D Hydrogel-Based Intestinal Epithelial-Fibroblast Co-Cultures for IBD Modeling

To establish a 3D co-culture system, we used HT-29 colorectal cancer-derived epithelial cells for their proliferative capacity, ease of culture, and inflammatory response to LPS [12]. IMR-90 fibroblasts were included to support epithelial growth via growth factors such as Wnt and EGF, promoting matrix deposition and vascularization within colonic organoids [15]. As hydrogel selection is critical for recapitulating the ECM in 3D cultures, we used a composite of alginate and gelatin to enhance biocompatibility, address mechanical fragility, and ensure reproducibility [18]. 3D cell culture models included single-cell culture models of either HT-29 intestinal epithelial cells or IMR-90 fibroblasts, as well as co-culture models containing HT-29 and IMR-90 cells at varying ratios of 1:0.5, 1:1, and 1:2, all seeded in A1G7 hydrogel. Assay evaluations of the culture systems revealed that the 3D co-culture model supports sustained cell metabolic activity, viability, and proliferation, as detailed in the following subsections.

#### 2.1.1. Cell Metabolic Activity

**Figure 1:**
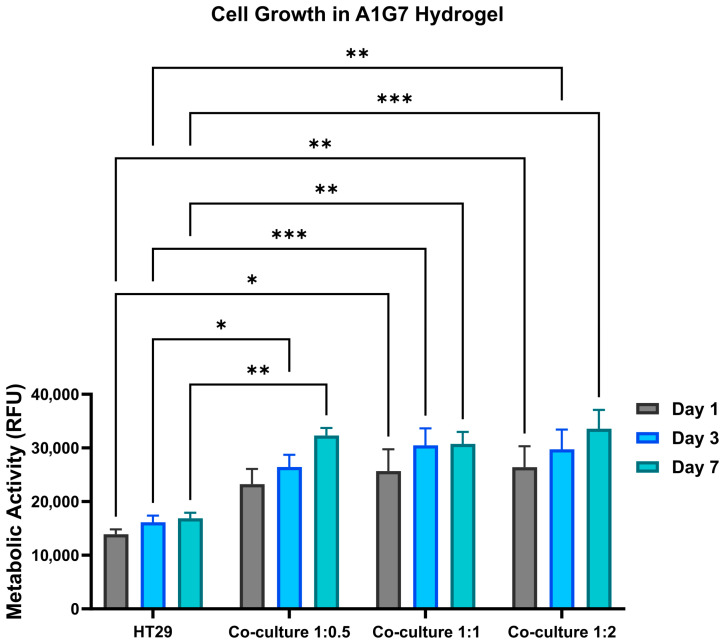
Quantification of metabolic activity using the AlamarBlue™ assay on Days 1, 3, and 7 of culture. Histogram depicting metabolic activity trends for HT-29 monocultures, IMR-90 fibroblast monocultures, and co-culture models at varying ratios (1:0.5, 1:1, and 1:2). Error bars represent mean ± SE (n = 3, * *p* < 0.5, ** *p* < 0.01, *** *p* < 0.001).

To determine metabolic activity, Alamar blue assays were performed. As shown in Figure 1, HT-29 mono-culture models demonstrated a consistent increase in metabolic activity, while IMR-90 fibroblast models displayed initially higher metabolic activity that stabilized on subsequent days. Co-culture models exhibited a 1.3- to 1.5-fold increase in metabolic activity, depending on the epithelial-to-fibroblast cell ratio over 7 days of culture. While all co-culture models exhibited high metabolic activity across ratios, the 1:0.5 ratio showed a progressive increase.

#### 2.1.2. Cell Viability

**Figure 2:**
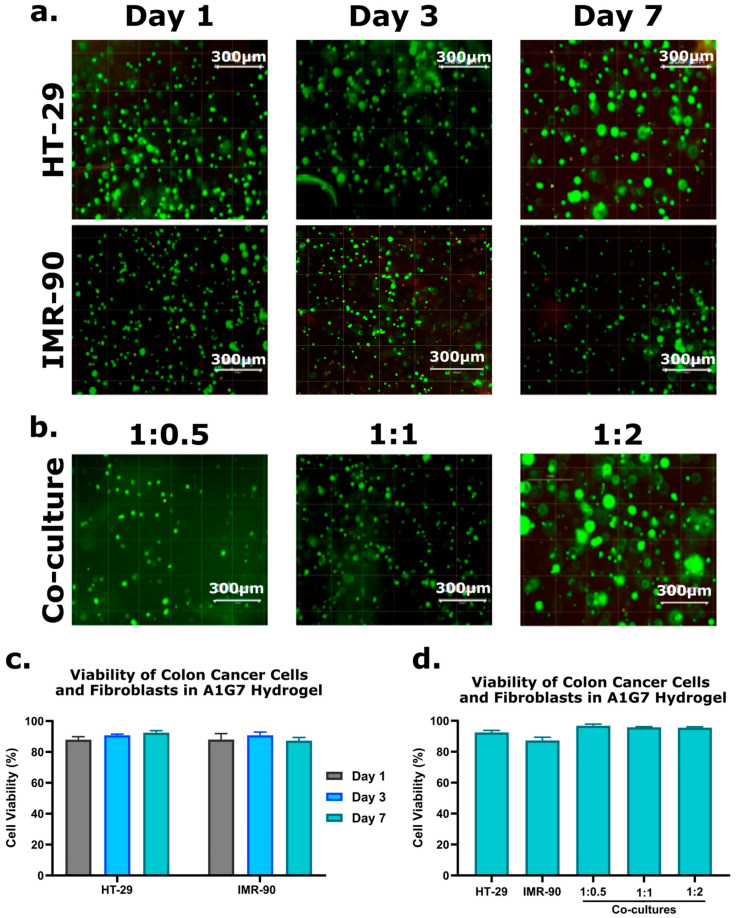
Cell viability analysis of 3D culture models using the Live/Dead™ assay. (**a**) Fluorescence microscopy images of HT-29 single cultures (**top row**) and IMR-90 fibroblast single cultures (**bottom row**) over 1, 3, and 7 days. (**b**) Fluorescence microscopy images of 3D co-culture models on Day 7, showing 1:0.5 (**left**), 1:1 (**middle**), and 1:2 (**right**) ratios. (**c**) Quantification of cell viability in 3D models at Days 1, 3, and 7. (**d**) Quantification of cell viability on Day 7 across single and co-culture models. Error bars represent mean ± SE (n = 3).

Live/Dead™ assay analysis indicated that cell viability remained consistently high (>92%) across all ratios of co-culture throughout the 7-day culture period, demonstrating the hydrogel’s supportive role in maintaining cell health. Representative fluorescence microscopy images in Figure 2a,b visually capture the predominance of live cells (green) with negligible numbers of dead cells (red) observed. Quantitative results of viability increase for HT-29 and IMR-90 monocultures (Figure 2c) and all co-culture ratios (Figure 2d) reinforce the model’s ability to replicate the natural cellular microenvironment [26,28].

#### 2.1.3. Cell Proliferation

**Figure 3:**
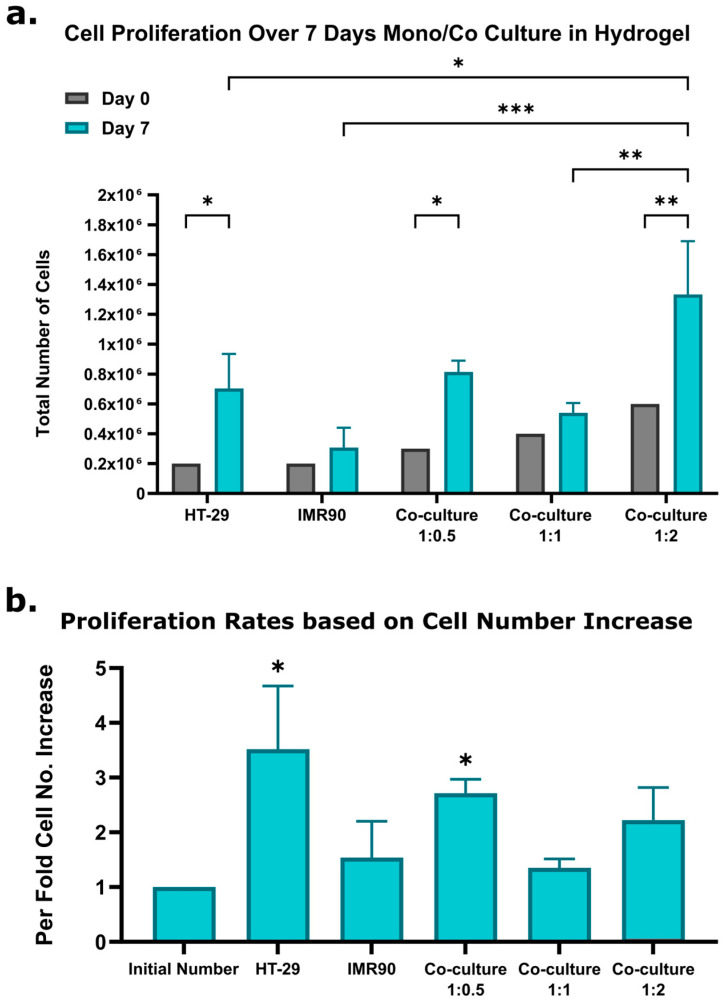
Cell proliferation in 3D culture models quantified using the Hoechst™ assay on Day 7. (**a**) Representative cell counts for HT-29 and IMR-90 single cultures, as well as co-culture models at varying fibroblast ratios (1:0.5, 1:1, and 1:2). (**b**) Proliferation rates calculated based on the estimated cell number increase for each model. Error bars represent mean ± SE (n = 3, * = *p* < 0.05, ** = *p* < 0.01, *** = *p* < 0.001).

To verify if increased metabolic activity is correlated with cell proliferation, cell numbers were quantified at day 7 (Figure 3a). Cell proliferation analysis showed that the HT-29 model had a 3.5 ± 1.15-fold increase, and the IMR-90 model had a 1.6 ± 0.66-fold increase in cell number. Figure 3b highlights the comparative per-fold increase across different models. The 3D co-culture models exhibited proliferation rates of 2.7 ± 0.25-fold (1:0.5 ratio), 1.3 ± 0.16-fold (1:1 ratio), and 2.2 ± 0.6-fold (1:2 ratio). Cell proliferation analysis further verified robust growth, with the 1:0.5 intestinal-to-fibroblast ratio yielding the highest proliferation rates. While the pericryptal fibroblast sheath-to-epithelial cell ratio in vivo is approximately 6.5:1 [17], we selected 1:0.5 for technical feasibility and its strong proliferative performance. The HT-29 cells are genetically different from the pericryptal cells, which may cause a difference in optimal co-culture ratios. Future studies may compare HT-29 cells to patient-derived intestinal cells in co-culture with stromal components.

### 2.2. Assessing Model Response to Inflammation and Anti-Inflammatory Treatments

Following the validation of cell viability, metabolic activity, and proliferation, we assessed the 3D co-culture model’s response to inflammatory and anti-inflammatory stimuli. Inflammation was induced using LPS (100 ng/mL) either (i) mixed directly with the cell-seeded hydrogel at the time of model formation or (ii) added to the culture medium after 24 h of incubation. For anti-inflammatory treatment, dexamethasone (10 µM) was introduced either together with LPS or 2 h after LPS exposure. Control models received neither LPS nor dexamethasone. TNF-α and IL-1β levels in the culture medium were quantified by ELISA 48 h after treatment.

**Figure 4:**
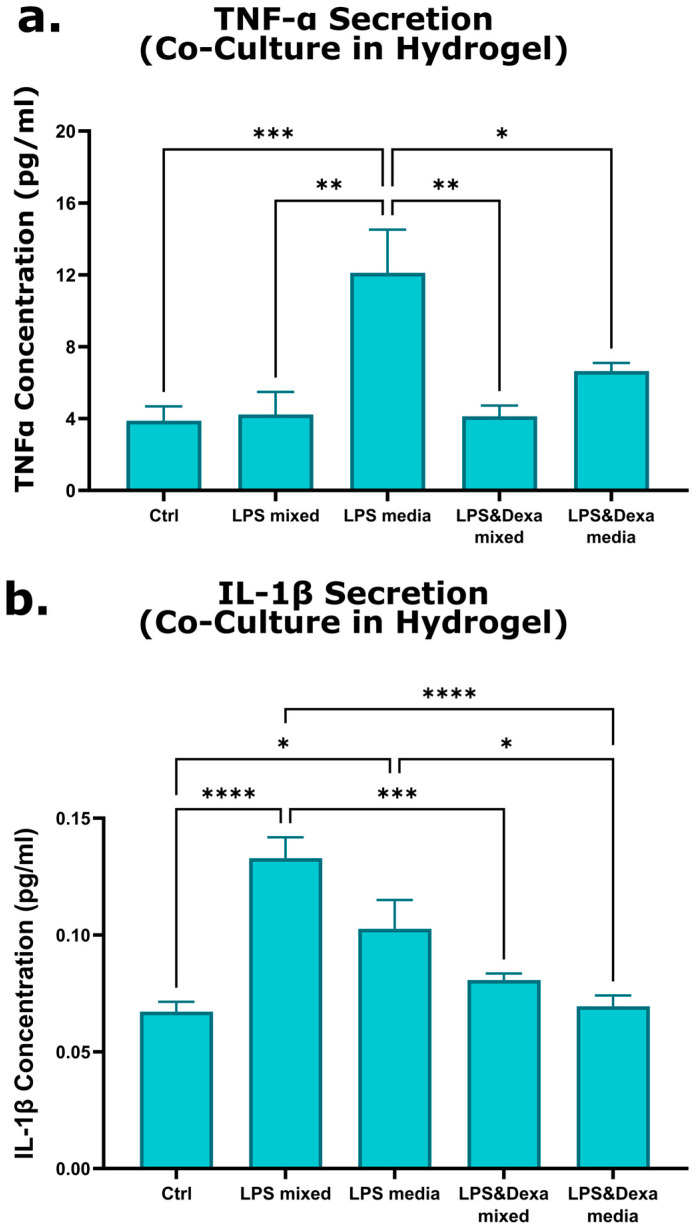
TNFα and IL-1β cytokine expression quantified by ELISA in 3D co-culture models. (**a**) TNFα concentration and (**b**) IL-1β concentration were measured in models where LPS was mixed directly with the cell-seeded hydrogel, LPS was added to the culture medium after 24 h of incubation, LPS was mixed with the hydrogel followed by dexamethasone treatment, LPS was added to the culture medium followed by dexamethasone treatment after 2 h, and neither LPS nor dexamethasone was administered (control). Culture medium was collected 48 h post-incubation, and cytokine expression levels were quantified using ELISA. Statistically significant differences (*p* < 0.005) were observed between LPS-exposed groups, dexamethasone-treated groups, and the control group. Error bars represent mean ± SE (n = 3, * *p* < 0.05, ** *p* < 0.01, *** *p* < 0.001, **** *p* < 0.0001).

LPS exposure increased cytokine production in all treatment conditions. As shown in Figure 4a, relative to the control group (TNF-α: 3.8 ± 0.8 pg/mL), TNF-α concentrations increased to 4.2 ± 1.2 pg/mL when LPS was mixed with the hydrogel and to 12.1 ± 2.4 pg/mL when LPS was added to the culture medium. As shown in Figure 4b, IL-1β concentrations increased from 0.07 ± 0.004 pg/mL in controls to 0.13 ± 0.009 pg/mL when LPS was mixed with the cells and to 0.1 ± 0.01 pg/mL when LPS was added to the medium. Dexamethasone treatment reduced cytokine levels in LPS-stimulated models. TNF-α decreased to 4.13 ± 0.6 pg/mL when co-administered with LPS in the hydrogel and to 6.65 ± 0.4 pg/mL when added after LPS exposure in the medium. IL-1β levels declined to 0.08 ± 0.003 pg/mL and to 0.07 ± 0.004 pg/mL under the same respective conditions.

### 2.3. Evaluation of 3D Interface Mesh Model Response to Inflammation and Anti-Inflammatory Treatment

**Figure 5:**
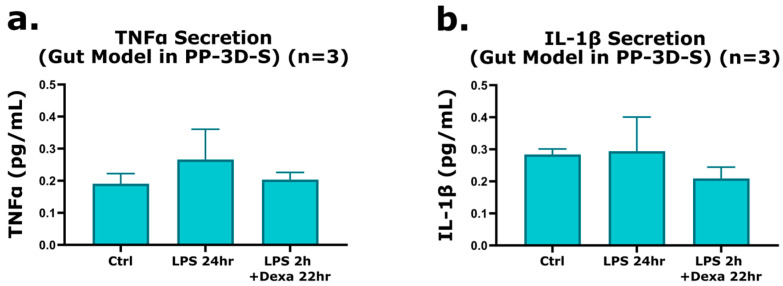
Quantification of TNFα and IL-1β expression by ELISA in the co-culture mesh model (PP-3D-S). (**a**) TNFα and (**b**) IL-1β concentrations were measured under three conditions: LPS treatment alone (100 ng/mL added after 24 h of incubation), LPS followed by Dexamethasone treatment (10 µM administered 2 h post-LPS for 22 h), and an untreated control group (no LPS or Dexamethasone). Error bars represent mean ± SE (n = 3).

To more accurately and closely replicate the physiological environment of human intestinal tissue, we further evaluated the response of the 3D interface mesh model to inflammatory and anti-inflammatory stimuli, demonstrating its applicability for modeling intestinal disease conditions. As outlined previously, the treatment protocol involved the use of lipopolysaccharide (LPS, 100 ng/mL) to induce inflammation and Dexamethasone (10 µM) as a well-established anti-inflammatory agent. ELISAs were used to quantify TNFα and IL-1β cytokine expression in response to LPS stimulation within the 3D co-culture mesh model. The treatment regimen applied was as follows: (i) LPS was added to the culture medium after 24 h of incubation to initiate inflammation; (ii) Dexamethasone was administered 2 h post-LPS exposure to evaluate its anti-inflammatory effects. Control groups were maintained in low-serum medium without any treatment. This experiment aimed to assess the model’s physiological relevance and responsiveness. As evidenced by the data presented in Figure 5a,b, LPS treatment noticeably induced TNFα and IL-1β expression in the 3D co-culture model compared to the control group. Specifically, TNFα levels increased by approximately 1.4 ± 0.09-fold (40%), while IL-1β expression exhibited a modest increase of 3.5% ± 0.1. Remarkably, co-administration of LPS and Dexamethasone resulted in a demonstrated reduction in both cytokines, with TNFα and IL-1β levels decreasing by 23.5% ± 0.02 and 29% ± 0.03, respectively. These findings highlight the anti-inflammatory effect of Dexamethasone in mitigating LPS-induced inflammation within the 3D gut model. These findings validate the responsiveness of our 3D co-culture model to both pro-inflammatory and anti-inflammatory treatments, emphasizing its utility as a physiologically relevant platform for drug screening and therapeutic evaluation.

### 2.4. Discussion

The aim of this study was to develop a reproducible 3D epithelial–stromal co-culture system capable of mimicking key features of the intestinal microenvironment and responding to inflammatory cues relevant to IBD. By combining HT-29 epithelial cells with IMR-90 fibroblasts in an alginate–gelatin hydrogel, we sought to establish a platform that bridges the gap between oversimplified 2D cultures and more complex but variable organoid-based systems. Overall, the model demonstrated sustained growth, high viability, active proliferation, and responsiveness to both pro-inflammatory and anti-inflammatory stimuli, supporting its feasibility for early-stage preclinical screening.

Hydrogel-based co-culture as a standardized alternative to organoids

Organoid models have transformed intestinal biology by enabling epithelial structures to self-organize into crypt- and villus-like domains, recapitulating differentiation, renewal, and patient-specific epithelial traits [32]. Despite their high physiological fidelity, organoids remain technically demanding, with batch-to-batch variability, and limited experimental access to stromal/immune compartments, restricting their utility for standardized assays or high-throughput screening [33,34]. In contrast, engineered 3D hydrogel-based systems offer a more controllable and reproducible platform while still capturing essential epithelial–stromal interactions. Unlike Matrigel, which suffers from lot-to-lot variability and limited biochemical definition, synthetic or bioinspired hydrogels provide a tunable microenvironment with more predictable biophysical and biochemical properties. This concept has been extensively articulated in the context of organoid engineering, where chemically defined “designer ECMs” have been proposed to improve reproducibility and experimental control [35]. Alginate–gelatin hydrogels, in particular, provide a tunable and biocompatible matrix with mechanical properties suitable for modeling epithelial–mesenchymal crosstalk, while avoiding the variability inherent to organoid-derived ECMs [34]. Importantly, the alginate–gelatin matrix was not developed here as a novel hydrogel formulation, but deliberately selected as a well-characterized and reproducible platform to enable controlled investigation of epithelial–stromal interactions and inflammatory responses.

The present co-culture model demonstrates stable viability, sustained proliferation, and functional responsiveness to both LPS-induced inflammation and dexamethasone-mediated suppression, supporting the notion that simplified 3D constructs can complement organoids in contexts where assay standardization, scalability, or controlled microenvironmental modulation are required [36]. Taken together, these features place hydrogel-based 3D co-cultures as an experimentally accessible intermediate between reductionist 2D monolayers and complex organoid or gut-on-chip platforms. In this sense, the present model can serve as a practical bridge for mechanistic studies and early-stage drug screening.

Cell growth and the influence of fibroblast-to-epithelial ratio

The choice of HT-29 epithelial cells and IMR-90 fibroblasts for establishing our first-generation 3D co-culture system was driven by practical considerations of reproducibility, handling, and cost-effectiveness. HT-29 cells are widely used for screening applications because they proliferate robustly and retain the ability to respond to inflammatory cues, including LPS-induced cytokine secretion, as previously reported [37]. IMR-90 fibroblasts, although not intestinal in origin, provide stromal support through matrix deposition and growth factor secretion, consistent with the known ability of fibroblasts to promote epithelial expansion in organoid and co-culture systems [38]. In addition, the cancer-derived origin of HT-29 cells represents a limitation in terms of physiological fidelity; however, their robust inflammatory responsiveness and widespread use in screening-oriented intestinal models make them suitable for first-line platform development prior to validation with primary or patient-derived epithelium.

In vivo, epithelial proliferation in the colon is sustained by a specialized pericryptal fibroblast sheath. Classic morphometric work in the murine colon reports approximately 124 fibroblasts per crypt, corresponding to an epithelial-to-stromal ratio of ~6.5:1 [17]. More recent studies using single-cell transcriptomics and niche-mapping further demonstrate that fibroblast subsets form a critical niche for epithelial stem cells [39]. However, these fibroblast populations are anatomically specialized and differ markedly from the homogeneous fibroblast lines typically used in engineered 3D models. For this reason, in vivo ratios cannot be directly translated into in vitro systems.

Accordingly, the 1:0.5 epithelial-to-fibroblast ratio reflects a technical optimization of the in vitro system rather than an attempt to directly reproduce physiological cellular proportions in vivo. Within our hydrogel model, the epithelial–stromal ratio of 1:0.5 yielded the highest metabolic and proliferative output. This empirically defined ratio likely reflects a balance between providing sufficient stromal support and avoiding excessive fibroblast density, which can alter ECM remodeling and nutrient availability. Similar empirical optimization of stromal ratios is commonly reported in engineered 3D intestinal models, reflecting the fact that fibroblast function in vitro is strongly influenced by culture conditions, cell-line origin, and matrix composition. Future iterations incorporating primary intestinal fibroblasts or mesenchymal subsets would enable a more physiologically relevant reconstruction of the epithelial niche.

Viability and proliferation within the alginate–gelatin hydrogel

Live/Dead imaging showed consistently high viability (>92%) at day 7 across all mono- and co-culture conditions, confirming that the alginate-gelatin (A1G7) hydrogel provides a biocompatible environment for epithelial and stromal cells. Quantification of DNA content further demonstrated that the increase in metabolic activity reflected genuine proliferation rather than isolated metabolic upregulation. These findings align with previous work showing that even non-adhesive alginate hydrogels can sustain the growth and maturation of intestinal organoids, with epithelial differentiation comparable to Matrigel-based cultures [40]. Recent advances in engineered matrices highlight that synthetic or hybrid hydrogels can be systematically tuned in stiffness, degradability, and ligand presentation, enabling more controlled modulation of epithelial growth and differentiation [41]. Such matrices have been shown to support the formation and expansion of intestinal organoids with improved reproducibility and reduced reliance on biologically derived ECM extracts, underscoring their suitability for standardized intestinal culture systems [42]. These considerations support the idea that the mechanical and biochemical properties of the A1G7 hydrogel are adequate for sustaining epithelial–stromal expansion in a reproducible manner.

In this context, the combination of a mechanically stable alginate network with gelatin-derived adhesive cues likely contributes to the observed maintenance of cell viability and proliferation by providing both structural support and permissive cell–matrix interactions. The widespread use of gelatin-alginate formulations in 3D biofabrication and epithelial tissue engineering further reinforces the suitability of this composite scaffold for reproducible, assay-compatible culture systems [43]. Despite these strengths, the static culture configuration imposes diffusion constraints and lacks the dynamic mechanical inputs present in vivo, representing an area for future refinement.

Inflammatory and anti-inflammatory responses

Exposure of the 3D co-culture system to LPS led to significant increases in TNF-α and IL-1β secretion, confirming that the epithelial-stromal construct can activate inflammatory pathways. This is consistent with previous work showing that HT-29 cells respond to LPS through TLR4-mediated signaling and secrete pro-inflammatory cytokines upon stimulation [38]. TNF-α and IL-1β were selected as representative early pro-inflammatory cytokines primarily driven by epithelial and stromal signaling, allowing inflammatory responsiveness to be assessed in the absence of immune cells. As such, the limited cytokine panel reflects a deliberate focus on epithelial–stromal contributions rather than immune-mediated inflammation. Although absolute cytokine concentrations remained low, their magnitude is consistent with previously reported immune-free 3D epithelial or epithelial–stromal systems. Importantly, the reproducible, stimulus-dependent modulation of cytokine levels, rather than their absolute concentration, was used as the primary functional readout to validate inflammatory activation and pharmacological sensitivity [44,45]. The mode of LPS delivery influenced the magnitude of cytokine induction: TNF-α secretion was more pronounced when LPS was added to the culture medium, whereas IL-1β increases were higher when LPS was incorporated directly within the hydrogel. These differences may reflect diffusion dynamics or spatial accessibility of TLR4-expressing cells. Comparable modality-dependent variations have been noted in other intestinal and gastric 3D systems exposed to LPS, where the physical context of the stimulus shapes downstream cytokine profiles [44,45].

Importantly, dexamethasone consistently reduced cytokine expression under all inflammatory conditions, demonstrating that the model is responsive to anti-inflammatory cues. Beyond its established glucocorticoid activity, dexamethasone has been shown to inhibit TLR4-associated pathways by targeting p38 MAPK signaling and limiting TNF-α-converting enzyme activity, thereby reducing cytokine release in LPS-stimulated cells [46]. Anti-inflammatory effects of dexamethasone have also been observed in 3D biomaterial systems, where controlled release from engineered scaffolds modulates local inflammatory activation [47]. These external findings reinforce the pharmacological fidelity of our model. Our observations also align with prior reports demonstrating that epithelial activation in response to microbial ligands can be modulated by cytokines or glucocorticoids, such as the inhibitory effects of IL-4 and IL-13 on LPS-driven epithelial responses [48].

Although the absence of immune cells limits the amplitude of cytokine production compared with immune-competent models, the system still showed a clear, reproducible increase in TNF-α and IL-1β in response to LPS, together with a consistent suppression by dexamethasone. This indicates that the epithelial–stromal compartment alone is sufficient to provide a measurable inflammatory readout. This supports the potential of the model for screening anti-inflammatory compounds. Future iterations integrating additional immune partners or expanding the cytokine panel would further enhance the biological relevance of the inflammatory responses captured in this system.

Interface mesh model: structural compartmentalization and reproducibility

The interface-based configuration, in which fibroblasts were seeded on a plasma-treated electrospun nanofibrous mesh and overlaid with an alginate–gelatin hydrogel containing epithelial cells, also exhibited robust TNF-α and IL-1β responses to LPS followed by suppression with dexamethasone. This confirms that epithelial–stromal crosstalk can be preserved across distinct architectural formats and suggests that the inflammatory readouts observed in the bulk hydrogel system are not specific to a single scaffold configuration. The use of an electrospun mesh as a stromal compartment is consistent with previous work showing that nanofibrous scaffolds can mimic key structural features of the native extracellular matrix and support the growth and differentiation of epithelial cells in three-dimensional culture [49,50]. In our model, fibroblasts are primarily confined within or on top of the fibrous layer, while epithelial cells are embedded in the hydrogel phase, resulting in a more defined separation between stromal and epithelial domains. Such compartmentalization approximates aspects of mucosal organization and may facilitate controlled studies of directional signaling across the interface. At the same time, established limitations of electrospun materials, such as restricted pore size and limited cell infiltration into densely packed meshes [51], highlight that further optimization of fiber architecture could refine cell distribution and matrix remodeling within the stromal compartment.

This architectural separation of stromal and epithelial compartments highlights how scaffold configuration, rather than hydrogel chemistry alone, can modulate epithelial–stromal crosstalk and inflammatory readouts. From a translational perspective, the mesh-based interface is inherently compatible with microphysiological and gut-on-a-chip platforms, in which fibrous scaffolds and hydrogel layers are integrated within perfused microfluidic devices to better reproduce tissue-tissue interfaces, mechanical cues, and luminal flow [52,53]. The ability of our interface model to recapitulate LPS-induced cytokine induction and its pharmacological suppression therefore provides a foundation for future integration into dynamic systems incorporating flow, peristalsis-like mechanical stimulation, immune components, or microbial consortia.

Limitations and future directions

Despite its strengths as an accessible and reproducible 3D system, this study has several limitations that shape its translational relevance. The reliance on immortalized epithelial and stromal cell lines represents a first constraint, as these lines do not fully capture the heterogeneity and region-specific functions of primary intestinal populations. Recent work has shown that fibroblast subtypes within the human intestine are highly diverse and contribute distinct niche signals [54]; incorporating patient-derived epithelial cells and physiologically matched stromal subsets would therefore increase biological fidelity. A second limitation is the absence of immune and vascular components. More complex 3D IBD models integrating macrophages have demonstrated stronger inflammatory outputs and more representative cytokine signatures [34], suggesting that adding defined immune populations could substantially broaden the range of measurable inflammatory responses in this system. Likewise, mechanical cues such as flow, deformation, and shear stress, poorly captured under static culture, play an important role in shaping epithelial behavior and barrier function [55]. Integrating this platform with microphysiological systems would help address these limitations.

From a translational perspective, while the present study focused on in vitro biological validation, alginate–gelatin hydrogels are inherently compatible with standardized preparation and short-term storage, which could facilitate their future adaptation to preclinical screening or personalized medicine workflows. However, systematic evaluation of long-term storage, sterilization strategies, and regulatory requirements would be required before considering medical device development, and remains an important direction for future work.

Finally, the relatively small number of biological replicates reduces statistical power, and longer-term studies will be needed to determine how stable epithelial-stromal interactions remain over time. Nonetheless, the modularity and reproducibility of this hydrogel-based approach provide a solid foundation for future developments. Combining this system with primary or patient-derived intestinal cells, as increasingly pursued for precision medicine applications [56], and expanding readouts to include barrier integrity, transcriptomic profiling, and multi-cytokine responses represent logical next steps. Such refinements will help position this model as a versatile tool for mechanistic studies and early-stage therapeutic screening in IBD.

## 3. Conclusions

Together, these results demonstrate that the 3D alginate–gelatin co-culture system provides a stable epithelial–stromal platform capable of sustaining growth, maintaining high viability, and generating measurable inflammatory responses that remain sensitive to pharmacological modulation. The combined influence of hydrogel composition, epithelial–fibroblast ratio, and mode of stimulation underscores the importance of controlled microenvironmental design in engineered intestinal models. By offering a reproducible and experimentally tractable alternative to organoid-based or fully microphysiological platforms, this system establishes a solid basis for mechanistic studies of epithelial–stromal interactions and early-stage testing of anti-inflammatory interventions. In the longer term, such controlled in vitro platforms may also support the development of more targeted therapeutic strategies, potentially improving treatment efficiency and reducing reliance on empirical therapeutic escalation. Its modular architecture also provides a clear path toward future iterations incorporating immune components, patient-derived cells, or dynamic perfusion for enhanced relevance to intestinal disease research.

## 4. Materials and Methods

### 4.1. Cell Culture, Seeding, and Preparation of 3D Mono-/Co-Culture Models

The HT-29 human colorectal adenocarcinoma cell line (American Type Culture Collection) and IMR-90 mCherry human fibroblast cell line (as described previously [57,58]) was utilized to generate mono- and co-culture models. Cells were cultured in high-glucose Dulbecco’s Modified Eagle Medium (DMEM; 4.5 g/L glucose) supplemented with 10% fetal bovine serum (FBS) and 1% penicillin/streptomycin (PS) at 37 °C in a humidified incubator with 5% CO_2_ (all from Gibco, Thermo Fisher, Toronto, ON, Canada). Cells were passaged at 80% confluency using 0.25% trypsin. To create 3D hydrogel models, an alginate–gelatin hydrogel (A1G7) was prepared by dissolving 1% (*w*/*v*) alginate in PBS at 80 °C under constant stirring, followed by the addition of 7% (*w*/*v*) gelatin at 40 °C. This alginate–gelatin composition was selected based on prior studies demonstrating that low-percentage alginate hydrogels provide a mechanically stable yet permissive 3D environment, while gelatin supplies cell-adhesive motifs supporting epithelial–stromal interactions. Calcium-mediated ionic crosslinking enables rapid and reproducible gelation under mild conditions, preserving cell viability and allowing controlled cell encapsulation. The hydrogel was sterilized under UV light for 24 h and stored at 4 °C until use. Before mixing with cells, the hydrogel was warmed to 37 °C, and 100 mM calcium chloride, CaCl_2_, (calcium chloride dihydrate, (C7902, Sigma, Oakville, ON, Canada)) was used as a cross-linking agent. For mono-cell models, 200,000 cells (either HT-29 or IMR-90) were suspended in 200 μL A1G7 hydrogel and seeded in 48-well plates. For co-culture models, HT-29 and IMR-90 cells were combined in 1:0.5, 1:1, and 1:2 intestinal cell-to-fibroblast ratios, with 300–600 μL hydrogel per well depending on the ratio. After cell seeding, 200 μL of CaCl_2_ was gently added on top of the hydrogel layer to cross-link the alginate part. Excess CaCl_2_ was aspirated after 10 min, and each well was rinsed with fresh medium to remove residual cross-linking agent. The models were maintained in DMEM with 10% FBS and 1% PS, and the medium was replaced every 3 days.

### 4.2. Inflammation Induction and Anti-Inflammatory Treatment

Inflammation was induced in the 3D co-culture models using lipopolysaccharide (LPS; 100 ng/mL) (Sigma-Aldrich). Two experimental conditions were applied: (1) LPS was added to the cells while mixing with hydrogel, and (2) LPS was introduced to the media of pre-cultured 3D models after 24 h. Control models were left untreated and DMEM media with 1% FBS and 1% PS were used for all experiments. For anti-inflammatory studies, the cells treated with LPS were subsequently exposed to dexamethasone (10 μM) (Sigma-Aldrich). Two setups (in DMEM media with 1% FBS and 1% PS) were utilized: (1) dexamethasone was added simultaneously with LPS, and (2) dexamethasone was applied 24 h post-LPS treatment. After 48 h, culture media were collected for cytokine analysis.

### 4.3. Inflammation Induction and Treatment Using the 3D Interface Model (PP-3D-S)

**Figure 6:**
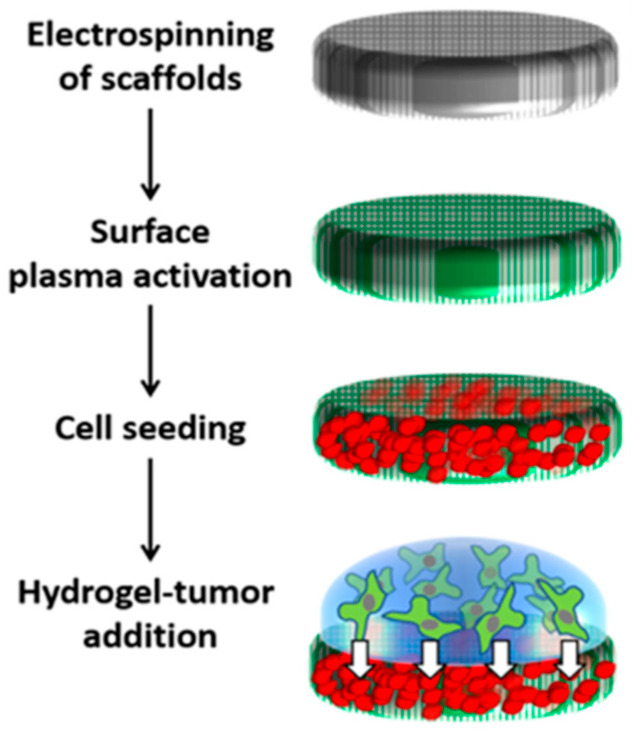
Schematic diagram of the 3D interface model (PP-3D-S) outlines a four-part process for creating a 3D cellular construct from top down: top, Electrospinning a 3D polylactic acid (PLA) electrospun scaffold. Second dow, Functionalizing the scaffold surface using plasma treatment. Third down, Seeding stromal cells onto the treated scaffold. Bottom, Applying a hydrogel layer containing pre-seeded tumor cells atop the 3D structure. Arrows indicate the consecutive additions to scaffold. Green color indicated plasma treatment, red are the fibroblasts, and green indicated the cells (HT29) suspended in the hydrogel.

To confirm the efficacy of inflammation treatment in different 3D in vitro models, we conducted a new experimental setup using a previously developed 3D interface model (PP-3D-S) [29,30,31] for comparison with the efficiency of the 3D hydrogel model utilized in this research. The PP-3D-S model (Figure 6) mimics a tumor microenvironment and consists of two primary components: (i) a stromal compartment which is a nanofibrous scaffold seeded with IMR-90 mCherry human fibroblasts and (ii) a 1% alginate-7% gelatin (A1G7) hydrogel embedded with HT-29 human colon tumor cells as tumor compartment. The scaffold was fabricated via the electrospinning of polylactic acid (PLA, NatureWorks 4032D, Plymouth, MN, USA), yielding randomly oriented fibers (600–800 nm diameter). To enhance the poor wettability and cell adhesion typical of synthetic polymeric scaffolds, the surface was modified using low-temperature, radiofrequency ammonia plasma treatment (NH_3_) (Air Liquide Canada Ltd., Montreal, QC, Canada). Treated scaffold disks (9 mm) were disinfected and placed in non-adherent 48-well plates (SARSTEDT AG & Co., Montreal, QC, Canada). Each disk was seeded with 200,000 IMR-90 mCherry fibroblasts and incubated overnight. Non-adherent cells were removed after 24 h. Subsequently, 100 µL of A1G7 hydrogel containing 200,000 HT-29 cells was applied atop the pre-seeded scaffold. Ionic crosslinking was induced by adding 200 µL of 100 mM calcium chloride, followed by the removal of the crosslinking agent, washing, and the addition of complete media. After 24 h of initial incubation, cells were subjected to inflammation treatment in DMEM media (1% FBS, 1% PS) under two distinct conditions: (i) 2 h LPS induction (100 ng/mL), followed by 22 h of treatment with a mixture of LPS (100 ng/mL) and Dexamethasone (10 μM); (ii) 24 h simultaneous treatment with LPS and Dexamethasone at the above concentrations. Following treatment, all culture media were collected for subsequent cytokine measurement using an ELISA.

### 4.4. Evaluation of 3D Models Performance

#### 4.4.1. Metabolic Activity Assay

Metabolic activity and proliferation of cells were assessed using the AlamarBlueTM assay (Thermo Fisher). Alamar Blue reagent was prepared at a 1:10 dilution in serum-free DMEM and added to each well (300 μL per well). Models were incubated at 37 °C for 6 h. Afterward, 100 μL of the medium from each well was transferred to a 96-well plate (black half-area; Corning, Terrebonne, QC, Canada), and fluorescence was measured at excitation/emission wavelengths of 540/590 nm using a Tecan Infinite M200 pro microplate reader (Tecan Trading, AG, Männedorf, Switzerland). Measurements were taken at days 1, 3, and 7.

#### 4.4.2. Cell Viability Assay

Cell viability was evaluated using the Live/Dead Viability/Cytotoxicity Assay (Thermo Fisher). After 7 days of culture, 200 μL of staining solution containing Calcien-AM and ethidium homodimer-1 in PBS at a dilution of 1:1000 and 1:500, respectively, was added to each well and incubated for 30 min at room temperature. Samples were imaged using an EVOS M5000 (Thermo Fisher, Burlington, ON, Canada) and images were analyzed with ImageJ (Version 1.54p, NIH open source) to calculate the percentage of viable cells.

#### 4.4.3. Cytokine Analysis

Pro-inflammatory cytokine levels (TNF-α and IL-1β) in the collected culture media were quantified using enzyme-linked immunosorbent assay (ELISA) kits (RayBiotech, Peachtree Corners, GA, USA). Assays were performed according to the manufacturer’s instructions. Absorbance was measured at excitation/emission wavelengths of 450/540 nm using a Tecan M200 Pro microplate reader (TECAN, Mannedorf, Switzerland) and cytokine concentrations were calculated from the standard curves.

#### 4.4.4. Cell Proliferation Assay

Cell proliferation was quantified using the Hoechst 33258 Assay (Thermo Fisher) according to the manufacturer’s instructions. After 7 days of culture, the cells in the 3D models were lysed with 4M guanidine hydrochloride buffer, GuHcl, (Sigma-Aldrich) for 48 h at 4 °C. All GuHcl extracts were diluted 10-fold before analysis and DNA content was measured at excitation/emission wavelengths of 352/460 nm using a Tecan M200 Pro microplate reader. DNA concentrations of each sample were interpolated from a calf-thymus DNA standard curve (Thermo Fisher).

### 4.5. Statistical Analysis

All experiments were conducted in triplicate and repeated three times to evaluate reproducibility. Results are expressed as mean ± standard error. Statistical analysis between two groups was performed using paired or unpaired *t*-tests. Multiple group comparisons were conducted using one- or two-way ANOVA, followed by multiple pairwise comparison tests for parametric data, with significance set at *p* < 0.05. Non-parametric data were analyzed using the Kruskal–Wallis test, with a subsequent Mann–Whitney U test applied for pairwise comparisons between independent groups. All analyses were conducted using GraphPad Prism (version 10.6.1).

## Data Availability

The data presented in this study are available from the corresponding author upon reasonable request.
